# The effects of simulated transport on the muscle characteristics of white-feathered end-of-cycle hens

**DOI:** 10.1016/j.psj.2021.101280

**Published:** 2021-05-23

**Authors:** C. Frerichs, K. Beaulac, T.G. Crowe, K. Schwean-Lardner

**Affiliations:** ⁎ Department of Animal and Poultry Science, University of Saskatchewan, Saskatoon, SK, Canada, S7N 5A8; † Department of Mechanical Engineering, University of Saskatchewan, Saskatoon, SK, Canada, S7N 5A9

**Keywords:** live shrink, muscle pH, muscle colour, water holding capacity, thermal stress

## Abstract

Transportation of end-of-cycle hens (**EOCH**) may result in birds’ experiencing metabolic stress, which changes muscle characteristics. This study evaluated the impacts of simulated transport on muscle characteristics of white-feathered EOCH (65–70 wk). The factorial arrangement included treatments of T/RH (-10°C uncontrolled RH [**-10**], 21°C with 30 [**21/30**] or 80% RH [**21/80**], 30°C with 30 [**30/30**] or 80% RH [**30/80**]), duration (4, 8, 12 h), and feather cover (105 well-feathered [**WF**], 105 poorly-feathered [**PF**]). A total of 210 hens/replicate/farm (farm=block; 3 total) were tested during the simulated transport. Crates (one/duration/replicate), divided in half for each feather cover (seven hens/side), were placed in a climate-controlled chamber. Prior to exposure, hens were fasted (6 h). BW was taken pre- and post-exposure, and the difference was calculated as live shrink. Post-exposure to the test conditions, birds were slaughtered and carcasses were analyzed for muscle characteristics. Data were analyzed as a randomized complete block design (farm of origin as block) with ANOVA (Proc Mixed, SAS 9.4; significance declared at *P* ≤ 0.05). Duration resulted in more weight loss for the birds (*P* < 0.01). Final pH measures (30 h post-mortem) were higher in hens exposed to -10 than 21/80, 30/30, and 30/80 and this difference was exacerbated with time (breast *P* < 0.01 and thigh *P* = 0.01). For muscle color, breast and thigh (both feather covers; *P* = 0.01) were darker in the -10 treatment while redness values were higher in EOCH exposed to this treatment (breast and thigh *P* < 0.01). Additionally, thigh muscle redness was higher in PF hens (*P* < 0.01). Thaw and cooking losses were impacted by T/RH and duration (thaw loss *P* = 0.03 and cook loss *P* = 0.04). Cook loss was also influenced by T/RH and feather cover with PF hen muscles losing less water during cooking in the -10 treatment (*P* = 0.01). Overall, the largest impact from transport was found in hens exposed for a longer duration to -10 antemortem compared to other treatments, demonstrating a significant impact on muscle characteristics from ante-mortem stress.

## INTRODUCTION

Muscle characteristics may signal physiological stress and its impact on meat quality ante-mortem in poultry. In terms of transportation, past research on muscle characteristics has predominantly focused on meat production species such as broilers or turkeys despite end-of-cycle-hens (**EOCH**) being an important component to the meat industry in terms of chicken broth and food by-products. EOCH pose a unique challenge because they are often metabolically exhausted from egg production demands during lay and may have poor feather coverage. High egg production may result in physiological strain on hens, resulting in a reduced body condition with limited energy reserves, leaving the birds vulnerable to fatigue during periods of heightened stress (Richards et al., 2012).

Stress during transport can lead to weight loss beyond that resulting from feed and water withdrawal in poultry (Knowles and Broom, 1990). The combination of minimal muscle mass, reduced feather cover (**FC**), and thermal demands during transport may trigger EOCH to rapidly use energy reserves for thermoregulation to maintain homeostasis (Weeks et al., 1997). Birds will use available blood glucose as an energy source until it becomes limited, then birds will mobilize glycogen reserves in the muscle tissue as well as fat for energy (Sherwood et al., 2013). Therefore, a longer transportation period requires more energy and leaves birds at risk to dehydration resulting in an increased body weight loss.

A well-established stressor during transport is thermal stress, which is known to influence the metabolism of muscles post-mortem from the activation of adrenal and physiological responses ante-mortem (Petracci et al., 2001). Heat stress can increase the rate of glycolysis, resulting in a rapid buildup of lactic acid in the muscle leading to a decline in muscle pH from decreased glycogen stores (Pearson and Young, 1989; Holm and Fletcher, 1997; Honikel, 2004). The decline in pH from hot temperature (**T**) exposure results in a pale, lighter muscle colouration (Babji et al., 1982) as a consequence of the denatured myoglobin from the increased rate of glycolysis (Dadgar et al., 2010). The paleness and decrease in overall pigmentation of muscles reflect an increased lightness (L*; Babji et al., 1982) and decreased redness (a*; Boulianne and King, 1995) which can be an indicator of pale, soft, exudative (**PSE**) muscles. The decrease in pH from heat stress also reduces water holding capacity (**WHC**) by altering the electrostatic forces between the myofiber contractile filaments of the protein, reducing the space between the filaments (Barbut, 1993; Honikel and Hamm, 1994; Sandercock et al., 2001). The decreased space and weakened electrostatic forces cause a rise in unbound water molecules, which lead to increased drip, thaw, and cook loss (Pearson and Young, 1989; Honikel and Hamm, 1994).

Cold stress can decrease or halt glycolysis, reducing lactic acid accumulation in the muscle and causing muscle pH to either remain neutral or rise (Lyon and Buhr, 1999). The higher pH from cold-T exposure provides muscles the ability to bind water, causing them to appear darker (lower L*), and redder (higher a*; Petracci et al., 2004; Dadgar et al., 2011). Dark colouration, reduced yellowness (b*), higher a*, firm texture, and dry appearance can be an indicator of dark, firm, dry (**DFD**) muscles (Dadgar et al., 2012a). The higher pH from cold stress or exhaustion causes the contractile proteins of the muscle to be further removed from their electrostatic points resulting in a net charge on the proteins and a larger intercellular space where water molecules may bind, providing muscles the ability to maintain more water (Honikel, 2004; Dadgar et al., 2010).

There have been few studies on the effects of duration (**D**) of transportation on muscle characteristics. Scholtyssek et al. (1977) reported an increase in live shrink with longer transportation D for broilers. Changes to muscle pH can influence other muscle quality characteristics such as color and WHC (Babji et al., 1982). Zhang et al. (2009) did not find an impact on muscle L* with different transport D. Bianchi et al. (2006) found short transport distances under 40 kilometers resulted in an increased breast muscle a*. Meanwhile, Zhang et al. (2009) reported muscles were yellower with increased transport D.

Prior studies have examined the impact of transportation on meat birds and have found that hot-T exposure causes lower muscle pH and WHC, and lighter muscle colouration, while cold-T exposure results in higher muscle pH and WHC, and darker and redder muscle colouration. However, analyses of RH have typically been absent in past research. Previous studies have demonstrated that changes to muscle characteristics were exacerbated by longer exposure, but limited research has been done on the impact of FC on muscle characteristics of EOCH. Therefore, this study has evaluated the impacts on muscle characteristics for well-feathered (**WF**) and poorly-feathered (**PF**) white strain EOCH exposed to heat and cold stress, at various D, which has not previously been studied.

## MATERIALS AND METHODS

The procedures for this experiment were approved by the University of Saskatchewan's Animal Care Committee and adhered to the Guide to the Care and Use of Experimental Animals set by the Canadian Council on Animal Care (2009). This experiment was part of a larger study that aimed to examine various effects of simulated transportation conditions on white-feathered EOCH. This portion of the experiment focuses on the impact to muscle characteristics.

### Experimental Design

The effects of exposing white-feathered EOCH to simulated transport were evaluated using 5 T/RH combinations (-10°C, uncontrolled RH (**-10**), 21°C 30% RH (**21/30**), 21°C 80% RH (**21/80**), 30°C 30% RH (**30/30**), 30°C 80% RH (**30/80**)), 3 simulated exposure D (4, 8, or 12 h), and 2 FC (WF and PF) in simulated chambers. Birds were not exposed to other transport stressors such as vibration. The study was arranged as a 5 × 3 × 2 factorial arrangement which was completed in 3 replicates with the farm of origin as block.

### Birds and Housing

Commercially raised white-feathered EOCH (Lohmann LSL-Lite; 65–70 wk of age; n = 630) were obtained from 3 local farms within a 120 kilometers radius of Saskatoon, Saskatchewan (210 hens/replicate (105 WF and 105 PF hens/replicate)). Hens were feather scored on farm using an adaptation of the 4-point feather scoring system described by Sarica et al. (2008), where scores 1 and 2 were classified as the PF hens (<50% FC) and scores 3 and 4 were WF hens (≥50% FC). Four areas of the body were evaluated: neck, back, breast, and wings. Once selected, EOCH were crated and transported in a climate-controlled van to the University of Saskatchewan 3 d prior to commencement of the experiment, to allow an acclimatization period following arrival. Hens were housed in 2 floor pens (3.9 × 3.0 m) with wheat straw litter, where conditions were maintained with a T of 15°C to 18°C and a RH between 40 and 60%. Feed (obtained from farm of origin) and water were provided ad libitum in 3 aluminum tube feeders (38 cm diameter) and 2 bell drinkers (36 cm diameter). The lighting program mimicked the farm of origin and was provided by 5 white light-emitting diode bulbs.

### Prior to Simulated Transport

Six hours prior to exposing birds to simulated transport, hens were fasted by being placed in one of 4 feed-withdrawal pens (21 hens/FC resulting in 7 hens/D); 1.2 × 1.3 m pen) with wheat straw litter and ad libitum access to an aluminum drinker pail (30 cm diameter). Following feed withdrawal, hens (7 WF and 7 PF) were randomly assigned to one T/RH combination and D. The EOCH were weighed (n = 7/replicate) and wing banded for identification before crating. Once crated, hens were given a 5-to-15-min lairage before being transported to the climate-controlled chambers (College of Engineering, University of Saskatchewan, Saskatoon, Canada).

### Simulated Transport

The climate-controlled chambers were monitored in real-time via a thermocouple and a multimeter (Omega HH509, Omega Engineering; Laval, Canada) and a RH sensor (HM1500LF, Measurement Specialities, Inc.; Toulouse, France). Upon arrival, hens were immediately moved from the temporary transport crates to modified experimental modular crates (density 53 kg/m^2^) that were divided in half (0.56 × 0.39m; 7 hens/FC/side/D). Modular crates were equipped with a T/RH data logger (iButton Hygrochron DS1923-#F5, Maxim Integrated; San Jose, CA) located on the middle partition at hen level, recording T/RH every minute. EOCH remained in the chamber for the allocated D (4, 8, or 12 h).

### Post-simulated Transport

Each crate was removed from the chamber after its respective D (4, 8, or 12 h) and immediately transferred to the processing room for further data collection (no lairage). EOCH were re-weighed, allowing calculation of live shrink. Five hens for each T/RH/FC/D were placed on shackles, stunned, and exsanguinated with an electric stunning knife (VS200, Midwest Processing Systems; Minneapolis, MN). Carcasses were scalded in a hot-water bath (68°C), mechanically plucked (Featherman Feather Plucker K7080; Featherman Equipment LCC; Suffolk County, NY) and manually eviscerated. The remaining 2 EOCH were manually cervically dislocated, and carcasses were disposed of.

After evisceration, an initial muscle pH reading was recorded (approximately 30 min post-mortem) by making a small vertical incision via scalpel on the upper right *pectoralis major* (breast) and right *iliotibial* (thigh) muscles and inserting a pH probe with a portable pH meter (Hanna H1 9025 pH meter, Hanna Instruments; Woonsocket, RI) and a T probe into the incision site. Next, carcasses were chilled for 1 h in an ice bath then placed on ice for 5 h in a refrigerator (4°C). After 6 h of chilling, the right *pectoralis* (major and minor) and *iliotibial* muscles were removed from the carcasses, weighed, and placed on a drip tray with a drip-lock pad, sealed with plastic wrap, and placed back in the refrigerator for 24 h. The left *pectoralis* (major and minor) muscles were also removed from the carcass, weighed, and placed in an individually labeled Ziploc freezer bag which were stored for 5 wk at -30°C until thaw and cook loss analyses were conducted.

After 24 h (30 h post-slaughter) in the refrigerator the right *pectoralis* (major and minor) and *iliotibial* muscles were reweighed and drip loss was calculated (drip loss (%) = ((initial muscle weight - final muscle weight) / initial weight) × 100). Then, final muscle pH and T were collected by making a second vertical incision adjacent to the initial site, repeating the method described above. Next, the muscles were allowed to bloom by exposing them to oxygen for 30 min. Before taking color readings, the Minolta color meter (CR-400, Konica Minolta Sensing Americas; Ramsey, NJ) was set to illuminate C and calibrated using a white colored tile. Then, 2 color readings were obtained, with the second reading at a 90° angle from the first to account for different muscle fiber orientation. The color readings were then exported to the computer and converted via SpectraMagicTM NX Software (Konica Minolta Sensing Americas, Inc., Ramsey, NJ) using illuminant source C and 2° to obtain L*, a*, and b* readings. The 2 sets of readings were averaged to obtain the final values.

After 5 wk post-slaughter, the left *pectorali*s (major and minor) muscles were thawed by storing them at 4°C for 24 h. The muscles were blotted with paper towel, weighed, and thaw loss was calculated (thaw loss (%) = ((initial muscle weight - final muscle weight) / initial weight) × 100). Muscles were then placed in a hot-water bath (80°C) and cooked until an internal T of 75°C (measured via thermocouple and multimeter). At an internal T of 75°C, muscles were left for an additional 5 min in the bath before removal and a final T reading was taken using a meat thermometer. Once muscles cooled to 40 to 50°C, they were blotted with paper towel, re-weighed, and cook loss was calculated (cook loss (%) = ((initial muscle weight - final muscle weight) / initial muscle weight) × 100).

### Statistical Analyses

Data analyses were conducted using SAS 9.4 (SAS 9.4, Cary, NC). The study was analyzed as a randomized complete block design, with farm of origin as block. The experimental unit for each parameter was a half crate. Data were checked for normality (PROC UNIVARIATE) and log transformed when not normally distributed before analyses. Treatment means and standard error of the means (SEM) were obtained using PROC MEANS, then an ANOVA was conducted (PROC MIXED). Means separation was conducted using Tukey's test and differences were considered significant at *P* ≤ 0.05.

## RESULTS

### Chamber and Crate Conditions

The actual climatic conditions (average crate T for each T/RH and D combination and average chamber T/RH combination) experienced by EOCH are reported in Table 1. The achieved chamber conditions were similar to the pre-determined T/RH combinations. Meanwhile crate conditions were typically higher than the targeted chamber T/RH combinations from nearby conspecifics within the crate.

**Table 1:** Average chamber conditions achieved and average crate conditions for temperature (T)/relative humidity (RH) combinations (-10°C uncontrolled RH (-10), 21°C 30% RH (21/30), 21°C 80% RH (21/80), 30°C 30% RH (30/30), and 30°C 80% RH (30/80)) during the entire duration of exposure.

	-10		21/30		21/80		30/30		30/80	
Crate	T (°C)	RH (%)	T (°C)	RH (%)	T (°C)	RH (%)	T (°C)	RH (%)	T (°C)	RH (%)
4 h	-1.55	55.14	25.89	44.97	26.02	68.06	34.27	32.00	34.55	69.82
8 h	4.44	48.30	35.64	43.10	27.15	64.71	34.04	31.59	33.42	71.56
12 h	-1.20	57.44	27.66	42.23	30.13	57.55	34.80	32.26	34.42	67.53
Chamber	-8.89	70.28	20.92	48.13	21.76	81.85	30.71	39.01	29.97	80.90

### Live Shrink

T/RH combinations and FC did not impact live shrink (%) of hens (Table 2). However, live shrink (%) rose with longer D, demonstrating the highest level after 12 h compared to 8 h, followed by 4 h (Table 2).

**Table 2:** Initial body weight (kg) and live shrink (%) parameters of white-feathered end-of-cycle hens with 2 feather covers (FC; well (WF) and poor (PF)) exposed to 5 temperature (T)/relative humidity (RH) combinations (-10°C uncontrolled RH (-10), 21°C 30% RH (21/30), 21°C 80% RH (21/80), 30°C 30% RH (30/30), and 30°C 80% RH (30/80)) for a duration (D) of 4, 8, or 12 h.

	Temperature/RH combinations						Duration				Feather Cover		
Parameter	-10	21/30	21/80	30/30	30/80	*P*-Value	4 h	8 h	12 h	*P*-Value	WF	PF	*P*-Value
Initial body weight (kg)	1.54	1.57	1.55	1.55	1.55	0.39	1.55	1.55	1.56	0.94	1.58^a^	1.52^b^	<0.01
Live shrink (%)	2.68	2.35	2.51	2.94	2.68	0.51	1.58^c^	2.65^b^	3.73^a^	<0.01	2.56	2.70	0.46


*P*-values for interactions	T/RHxD			T/RHxFC		DxFC			T/RHxDxFC		SEM^1^		
Initial body weight	0.52			0.12		0.44			0.09		0.008		
Live shrink	0.74			0.06		0.85			0.15		0.153		

a,b,c Means within a main effect with different superscripts are significantly different (*P* ≤ 0.05).

1 Standard error of the mean.

### Breast and Thigh Muscle pH

Breast Muscle pH: There was an interaction between T/RH combinations and D for final breast muscle pH (Tables 3 and 4). Final breast muscle pH was highest for birds exposed to -10 for 12 h compared to those exposed to -10 for 8 h, followed by all other T/RH combinations at all D exposures (Table 3). Initial breast muscle pH was impacted by T/RH combinations with hens exposed to -10 having a higher pH than those exposed to 30/30 combination (Table 4). No impact was observed for FC on initial or final breast muscle pH or D for initial breast muscle pH (Table 4).

**Table 3:** Muscle characteristics: Interactions between temperature/relative humidity combinations and duration of exposure for white-feathered end-of-cycle hens exposed to 5 temperature/relative humidity combinations (-10°C uncontrolled RH (-10), 21°C 30% RH (21/30), 21°C 80% RH (21/80), 30°C 30% RH (30/30), and 30°C 80% RH (30/80)) for a duration of 4, 8, or 12 h.

Parameter	-10	21/30	21/80	30/30	30/80
Thaw loss (%)					
4 h	0.85^c^	2.34^b^	3.42^ab^	2.78^b^	2.64^b^
8 h	2.03^bc^	3.62^ab^	4.63^ab^	1.92^bc^	2.36^b^
12 h	0.77^c^	2.26^b^	3.26^ab^	4.66^a^	2.29^b^
Cook loss (%)					
4 h	15.93^bc^	18.16^ab^	21.07^a^	19.71^a^	18.56^ab^
8 h	17.73^ab^	21.00^a^	20.32^a^	20.54^a^	19.97^a^
12 h	12.65^c^	19.92^a^	19.99^a^	17.92^ab^	18.33^ab^
Final breast pH					
4 h	6.21^bc^	5.95^cd^	5.87^d^	5.86^d^	5.84^d^
8 h	6.32^b^	5.85^d^	5.92^d^	5.89^d^	5.79^d^
12 h	6.80^a^	5.95^cd^	5.90^d^	5.87^d^	5.90^d^
Initial thigh pH					
4 h	6.92^ab^	6.69^bc^	6.61^bc^	6.64^bc^	6.68^bc^
8 h	6.90abc	6.51^bc^	6.46^c^	6.38^c^	6.74^bc^
12 h	7.29^a^	6.50^bc^	6.58^bc^	6.49^bc^	6.58^bc^
Final thigh pH					
4 h	6.70^b^	6.33^c^	6.21^c^	6.16^c^	6.27^c^
8 h	6.81^ab^	6.22^c^	6.23^c^	6.21^c^	6.17^c^
12 h	7.08^a^	6.21^c^	6.21^c^	6.17^c^	6.14^c^

a,b,c,d Means with different superscripts within a parameter are significantly different (*P* ≤ 0.05).

**Table 4:** Breast muscle characteristics of white-feathered end-of-cycle hens with 2 feather covers (FC; well (WF) and poor (PF)) exposed to 5 temperature (T)/relative humidity (RH) combinations (-10°C uncontrolled RH (-10), 21°C 30% RH (21/30), 21°C 80% RH (21/80), 30°C 30% RH (30/30), and 30°C 80% RH (30/80)) for a duration (D) of 4, 8, or 12 h.

	Temperature/RH combinations						Duration				Feather cover			
Parameter^1^	-10	21/30	21/80	30/30	30/80	*P*-value	4 h	8 h	12 h	*P*-value	WF		PF	*P*-value
Drip loss (%)	0.41	0.60	0.47	0.85	1.00	0.16	0.60	0.85	0.62	0.64		0.74	0.63	0.38
Thaw loss (%)	1.15^b^	2.74^ab^	3.77^a^	3.12^a^	2.38^ab^	<0.01	2.46	3.01	2.86	0.38		2.45	3.11	0.10
Cook loss (%)	15.53^b^	19.69^a^	20.46^a^	19.39^a^	18.88^a^	<0.01	18.78^ab^	20.15^a^	18.33^b^	<0.01		19.12	19.04	0.10
L*	43.02^b^	46.78^a^	48.79^a^	47.22^a^	47.41^a^	<0.01	47.00	47.50	46.48	0.11		46.87	47.14	0.60
a*	9.16^a^	8.15^b^	7.98^b^	8.17^b^	8.54^ab^	<0.01	8.40	8.11	8.39	0.34		8.31	8.29	0.40
b*	-1.52^c^	-0.62^b^	0.57^a^	-0.21^ab^	-0.24^ab^	<0.01	-0.07^a^	-0.24^ab^	-0.66^b^	<0.01		-0.34	-0.29	0.51
Initial pH	6.74^a^	6.50^ab^	6.49^ab^	6.34^b^	6.48^ab^	<0.01	6.54	6.45	6.48	0.27		6.48	6.51	0.53
Final pH	6.40^a^	5.92^b^	5.90^b^	5.87^b^	5.84^b^	<0.01	5.94^b^	5.91^b^	6.01^a^	<0.01		5.96	5.94	0.22


*P*-values for interactions^1^		T/RHxD		T/RHxFC		DxFC		T/RHxDxFC				SEM^2^		
Drip loss (%)		0.71		0.67		0.70		0.97				0.090		
Thaw loss (%)		0.03		0.51		0.84		0.85				0.183		
Cook loss (%)		0.04		0.01		0.74		0.17				0.285		
L*		0.76		0.38		0.88		0.84				0.307		
a*		0.50		0.36		0.15		0.23				0.138		
b*		0.32		0.35		0.43		0.77				0.133		
Initial pH		0.51		0.89		0.91		0.97				0.027		
Final pH		<0.01		0.17		0.33		0.87				0.026		

a,b,c Means within a main effect with different superscripts are significantly different (*P* ≤ 0.05).

1 Drip, thaw, and cook losses are measured in %; L* = Lightness; a* = redness; b* = yellowness.

2 Standard error of the mean.

Thigh Muscle pH: T/RH in combination with D had interactive effects for both initial and final thigh muscle pH (Tables 3 and 5). Initial thigh muscle pH was higher for birds exposed to -10 for 12 h compared to those exposed to 21/30, 21/80, 30/30, and 30/80 at all D (Table 3). Final thigh muscle pH was highest for hens exposed to -10 for 12 h compared to the -10 for 4 h, followed by all other T/RH combinations across all D (Table 3). FC had no impact on initial or final thigh muscle pH for the birds (Table 5).

**Table 5:** Thigh muscle characteristics of white-feathered end-of-cycle hens with 2 feather covers (FC; well (WF) and poor (PF)) exposed to 5 temperature (T)/relative humidity (RH) combinations (-10°C uncontrolled RH (-10), 21°C 30% RH (21/30), 21°C 80% RH (21/80), 30°C 30% RH (30/30), and 30°C 80% RH (30/80)) for a duration (D) of 4, 8, or 12 h.

	Temperature/RH combinations						Duration				Feather cover		
Parameter^1^	-10	21/30	21/80	30/30	30/80	*P*-Value	4 h	8 h	12 h	*P*-Value	WF	PF	*P-*Value
Drip loss	0.47	0.47	0.55	0.63	0.11	0.53	0.48	0.30	0.55	0.58	0.31	0.59	0.30
L*	41.87^c^	47.60^b^	49.87^a^	49.0^ab^	47.87^b^	<0.01	47.37	47.98	47.93	0.93	47.43	48.10	0.74
a*	7.81^a^	7.03^b^	6.60^b^	6.91^b^	7.33^ab^	<0.01	7.08	7.05	7.00	0.99	6.76^b^	7.34^a^	<0.01
b*	-6.07^c^	-3.47^b^	-2.97^ab^	-2.30^a^	-3.26^b^	<0.01	-3.64	-3.43	-3.15	0.74	-3.72	-3.09	0.27
Initial pH	7.01^a^	6.57^b^	6.55^b^	6.50^b^	6.67^b^	<0.01	6.70	6.57	6.62	0.12	6.64	6.62	0.62
Final pH	6.83^a^	6.25^b^	6.22^b^	6.18^b^	6.19^b^	<0.01	6.32	6.27	6.28	0.52	6.32	6.26	0.94


*P*-values for interactions^1^		T/RHxD		T/RHxFC		DxFC			T/RHxDxFC		SEM^2^		
Drip loss		0.56		0.56		0.51			0.63		0.103		
L*		0.43		0.01		0.91			0.83		0.354		
a*		0.81		0.33		0.88			0.38		0.105		
b*		0.97		0.35		0.80			0.99		0.173		
Initial pH		0.05		0.90		0.77			0.94		0.028		
Final pH		0.01		0.06		0.67			0.95		0.029		

a,b,c Means within a main effect with different superscripts are significantly different (*P* ≤ 0.05).

1 Drip loss measured in %; L* = Lightness; a* = redness; b* = yellowness.

2 Standard error of the mean.

### Muscle Color

Breast Muscle Lightness (L*): D and FC did not affect breast muscle lightness (Table 4). The breast muscle lightness was impacted by T/RH, with the EOCH exposed to -10 having a lower breast muscle L* compared to those exposed to other T/RH (Table 4).

Thigh Muscle Lightness (L*): There was an interaction between T/RH combinations and FC for thigh muscle lightness (Tables 5 and 6). Hens of both FC exposed to the -10 combination had a lower thigh muscle L* compared to EOCH of both FC exposed to all other T/RH combinations (Table 6). There was no effect by D on birds’ thigh muscle L* (Table 5).

**Table 6:** Muscle characteristics: Interactions between temperature/relative humidity combinations and feather cover for white-feathered end-of-cycle hens with 2 feather covers (FC; well (WF) and poor (PF)) exposed to 5 temperature/relative humidity combinations (-10°C uncontrolled RH (-10), 21°C 30% RH (21/30), 21°C 80% RH (21/80), 30°C 30% RH (30/30), and 30°C 80% RH (30/80)).

Parameter	-10	21/30	21/80	30/30	30/80
Cook Loss (%)					
WF	16.99^b^	19.48^ab^	20.41^a^	19.79^ab^	18.46^ab^
PF	12.97^c^	19.91^ab^	20.51^a^	18.99^ab^	19.44^ab^
Thigh Lightness (L*)					
WF	43.30^b^	47.87^a^	49.18^a^	48.46^a^	47.41^a^
PF	39.35^b^	47.52^a^	50.55^a^	49.74^a^	48.46^a^

a,b,c Means within a parameter with different superscripts are significantly different (*P* ≤ 0.05).

Breast Muscle Redness (a*): There was an influence on breast muscle redness from T/RH combinations with the hens exposed to the -10 combination having a higher breast muscle a* compared to those exposed to 21/30, 21/80, and 30/30 combinations (Table 4). No D or FC impact was reported for breast muscle a* (Table 4).

Thigh Muscle Redness (a*): Thigh muscle redness was impacted by T/RH combinations with birds exposed to the -10 combination having a higher thigh muscle a* compared to those exposed to 21/30, 21/80, and 30/30 combinations (Table 5). Furthermore, PF hens had a higher thigh muscle a* compared to WF birds (Table 5). There was no effect by D on thigh muscle a* (Table 5).

Breast Muscle Yellowness (b*): Breast muscle yellowness was impacted by T/RH combinations with birds exposed to 21/80 combination being more yellow than the 21/30 combination, followed by the -10 combination (Table 4). There was also an effect on D for breast muscle b* with birds exposed for 4 h being higher than those exposed for 12 h (Table 4). No effect was reported for FC on breast muscle b* (Table 4).

Thigh Muscle Yellowness (b*): An effect was observed for T/RH combination on thigh muscle yellowness, with birds exposed to the 30/30 combination being more yellow than those exposed to 21/30 and 30/80 combinations, followed by the -10 combination (Table 5). There was no impact from D or FC on thigh muscle b* (Table 5).

### Drip, Thaw, and Cook Loss

Drip Loss (%): No T/RH, D or FC effect was noted for breast or thigh muscle drip loss (Tables 4 and 5).

Thaw Loss (%): An interaction was observed for breast muscle thaw loss between T/RH combinations and D (Tables 3 and 4). Breast muscle thaw loss was greatest in hens exposed for 12 h to 30/30 compared to those exposed for 4 h to 30/30, all D at 30/80, and the 4 and 12 h exposure at 21/30, followed by EOCH exposed for 4 and 12 h at -10 (Table 3). There was no effect on FC for breast muscle thaw loss (Table 4).

Cook Loss (%): There was an interaction for breast muscle cook loss between T/RH combinations and D (Tables 3 and 4). Breast muscle cook loss was greatest for hens exposed for 8 and 12 h to 21/30, all D at 21/80, 4 and 8 h at 30/30, and 8h at 30/80 compared to those exposed for 4 and 12 h to -10 (Table 3). A second interaction was observed for breast muscle cook loss between T/RH combinations and FC (Tables 4 and 6). Birds of both FC exposed to 21/80 had a higher breast muscle cook loss compared to WF hens at -10, followed by the PF EOCH in the -10 (Table 6).

## DISCUSSION

During transportation, interior T/RH combination of a transport trailer can impact EOCH thermoregulatory responses. Past research has found that poultry experience a microclimate within transport trailers which is attributed to a lack of air movement, heat generation and respiration from conspecifics (Knezacek et al., 2010). In this study, the actual average chamber T/RH combinations were comparable to the targeted T/RH combinations. Meanwhile, the T/RH combinations recorded among the EOCH in the crates were warmer and generally more humid than the pre-determined T/RH combinations.

Transportation is a stressful event for poultry. Stressors such as extreme T/RH combinations, longer D, and poor FC can influence weight loss (Knowles and Broom, 1990; Petracci et al., 2001; Jacobs et al., 2016a, b, c) by causing EOCH to implement thermoregulatory mechanisms to cope, which quickly deplete the hens’ limited energy reserves. The data from the current study demonstrates that longer exposures resulted in an increase in the percentage loss, expressed as live shrink. Scholtyssek et al. (1977) reported similar findings with broilers transported for 4.5 h having a weight loss of 3.1% compared to birds transported for 3 h or 1.5 h with a weight loss of 2.3% for both. Holm and Fletcher (1997) demonstrated a similar exposure D to the current study, with broilers exposed for 12 h to 29°C. This resulted in a 6.20% loss in live weight compared to 3.75% and 3.82% for those exposed to 7°C and 18°C, respectively. Similarly, at a higher T, Petracci et al. (2001) found a 5.67% live shrink loss for broilers exposed for 12 h to 34°C compared to birds exposed to 24°C and 29.5°C, which had a live shrink loss of 3.16% and 3.87%, respectively.

The impact of thermal stress can be exacerbated with length of exposure resulting in alterations to muscle pH post-mortem. Dadgar et al. (2010) demonstrated a higher breast muscle pH for broilers exposed for 3 to 4 h to T below 0°C (pH = 5.98) compared to T between 0 to 20°C (pH = 5.91) and T above 20°C (pH = 5.84). Similarly, Dadgar et al. (2012b) found broilers had higher thigh muscle pH when exposed for 3 h to T below -11°C compared to birds exposed to T between 0 to -11°C, followed by those exposed to 22°C. The data in the present study demonstrated a similar response to the cold, with EOCH exposed to the cold T/RH combination for an extended D resulting in a higher pH in both the breast and thigh muscles (except initial breast muscle pH which only had a T/RH combination effect).

The changes in pH values due to thermal stress can affect other muscle characteristics such as muscle color (Barbut, 1997; Dadgar et al. 2010, 2011). In addition to pH, muscle color such as L* values ≥53 or a* values of ≤46 can be indicators of PSE and DFD muscle characteristics, respectively (Mckee and Sams, 1997; Bianchi et al., 2005; Dadgar et al., 2012a, b). The present study found that hens of both FC exposed ante-mortem to the cold T/RH combination had darker thigh muscle colouration (lower L*). The darker thigh muscle colouration resulted in higher a* when exposed to the cold T/RH combination and particularly impacted PF hens which had a redder appearance (higher a*) compared to the WF birds. Dadgar et al. (2012b) had similar findings with broilers exposed to T below 0°C resulting in thigh muscles that were darker and redder. Hen breast muscle color in this study demonstrated similar findings. The birds exposed to the cold T/RH combination ante-mortem had darker, redder breast muscle colouration (lower L* and higher a*). The impact of cold stress on breast muscles has been reported in Dadgar et al. (2010) where broilers exposed to T below 0°C for 3 to 4 h were darker and redder than those exposed to T above 0°C. The present study indicates that breast and thigh muscles of EOCH exposed to cold T/RH combinations can be characterized based on colouration as DFD muscles. Meanwhile, the other T/RH combinations, particularly the hot exposures did not demonstrate the development of PSE characteristics in the breast or thigh muscles despite the decline in pH values.

Past research has reported limited change to muscle yellowness (b*), even when other muscle color values such as lightness (L*) have been altered (Fletcher et al., 2000; Van Laack et al., 2000; Bianchi et al., 2005). Holm and Fletcher (1997) demonstrated that a higher T exposure of 29°C for 12 h resulted in an increase broiler breast muscle yellowness compared to those exposed to 18°C and 7°C ante-mortem. The present study also found T/RH combinations had an impact on EOCH muscle yellowness. Hens exposed to neutral T, high RH combination ante-mortem had the greatest impact on the birds’ breast muscle yellowness, while hot T, low RH combination ante-mortem impacted birds thigh muscle yellowness. The extended exposure resulted in a decrease of EOCH breast muscle yellowness.

Muscle pH also plays a significant role in WHC of the muscle by impacting the isoelectric forces between the protein filaments and changing the space between the proteins (Offer and Knight, 1988; Pearson and Young, 1989). Based on the significant differences for breast and thigh muscle pH in this study, it would have been anticipated that WHC values, drip loss, thaw loss, and cook loss would have also been impacted in the extreme T/RH combinations at increased D. However, there was no influence from treatment exposure on EOCH breast or thigh muscle drip loss. Dadgar et al. (2010) demonstrated similar findings with exposure to T between -27°C to 11°C for 3 to 4h not impacting broiler meat drip loss. Dadgar et al. (2011) explained that the lack of significant differences in drip loss may be attributed to measuring drip loss at 24 h instead of +48 h. When measuring drip loss at 48 h, Sandercock et al. (2001) reported significant results for broilers exposed to 32.5°C with an RH of 67.1%. The current study found a thaw loss reduction for EOCH exposed to cold T/RH combinations for the shortest and longest D. Similarly, Dadgar et al. (2012a, b) found that broiler breast and thigh muscles from cold stressed birds exposed for 3 to 4 h had a smaller thaw loss. EOCH in the current study exposed to the cold T/RH combination for the longest D ante-mortem demonstrated minimal cook loss. Other studies evaluating the impact of thermal stress and length of exposure on cook loss have been inconclusive. Holms and Fletcher (1997) reported a decline in cook loss for broilers exposed for 12 h to high T, while Dadgar et al. (2010) found no impact to cook loss for broilers during a 3 to 4 h exposure to T ranging from -27°C to 11°C ante-mortem. The present study reported an impact on EOCH breast muscle cook loss with WF and PF hens reacting differently to T/RH combinations. PF hens exposed to the cold T/RH combination had limited water loss during the cooking process. To the authors knowledge there are no other studies that have evaluated the relationship between T/RH combinations and FC on cook loss.

Overall, this study demonstrated that white-feathered EOCH had limited changes in muscle characteristics when exposed to heat stress over an extended period regardless of FC. Hot exposure increased the rate of glycolysis, leading to a rapid buildup of lactic acid, which resulted in lower pH levels. The increased rate of glycolysis resulted in denaturation of myoglobin leading to paler, lighter muscles and weakening of electrostatic forces resulting in higher water loss. However, EOCH exposed to cold stress demonstrated significant changes to muscle characteristics especially during extended exposure. Additionally, FC also influenced breast muscle cook loss and thigh muscle lightness in the cold conditions. This study found that longer cold exposure decreased glycolysis, reducing lactic acid accumulation, which resulted in elevated pH levels. Higher pH levels result in muscles binding water, creating a darker and redder muscle that holds more water. Extended exposure and poor feather coverage are 2 variables which exacerbate the muscle characteristic effects of cold stress on EOCH.
